# Crystal structure of 1,3-bis­(3-*tert*-butyl-2-hy­droxy-5-methyl­benz­yl)-1,3-diazinan-5-ol monohydrate

**DOI:** 10.1107/S2056989016013645

**Published:** 2016-08-31

**Authors:** Augusto Rivera, Ingrid Miranda-Carvajal, Jaime Ríos-Motta, Michael Bolte

**Affiliations:** a Universidad Nacional de Colombia, Sede Bogotá, Facultad de Ciencias, Departamento de Química, Cra 30 No. 45-03, Bogotá, Código Postal 111321, Colombia; b Institut für Anorganische Chemie, J. W. Goethe-Universität Frankfurt, Max-von Laue-Strasse 7, 60438 Frankfurt/Main, Germany

**Keywords:** crystal structure, hexa­hydro­pyrimidine, 1,3-diazinane, hydrogen bond

## Abstract

In the title hydrate, C_28_H_42_N_2_O_3_·H_2_O, the central 1,3-diazinan-5-ol ring adopts a chair conformation with the two benzyl substituents equatorial and the lone pairs of the N atoms axial. The dihedral angle between the aromatic rings is 19.68 (38)°. There are two intra­molecular O—H⋯N hydrogen bonds, each generating an *S*(6) ring motif. In the crystal, classical O—H⋯O hydrogen bonds connect the 1,3-diazinane and water mol­ecules into columns extending along the *b* axis. The crystal structure was refined as a two-component twin with a fractional contribution to the minor domain of 0.0922 (18).

## Abstract 2

The asymmetric unit comprises one 1,3-bis­(3-*tert*-butyl-2-hy­droxy-5-methyl­benz­yl)-1,3-diazinan-5-ol mol­ecule and one water mol­ecule. The two mol­ecular components are held together through an O—H⋯O hydrogen bond.

## Chemical context   

Current research of our group is directed toward the synthesis of cyclic aminals with conformational inter­est, which may have the structural requirement for hydrogen-bonded inter­actions. Obvious targets are the 5-hy­droxy-1,3-diazinanes because a hydroxyl group in the six-membered 1,3-di­aza­cyclic ring may alter the conformational preferences resulting from the inter­actions of the hydroxyl group and the endocyclic nitro­gen atoms (Salzner, 1995 ▸). We gradually realized that the structural features of this class of compounds are much more complex than previously believed and defined. Thus, we intend to use X-ray investigations to complement the information on conformational preferences and electronic parameters of 5-hy­droxy-1,3-diazinanes obtained using NMR chemical shift data, spin–spin coupling constants, and their NOESY spectra.
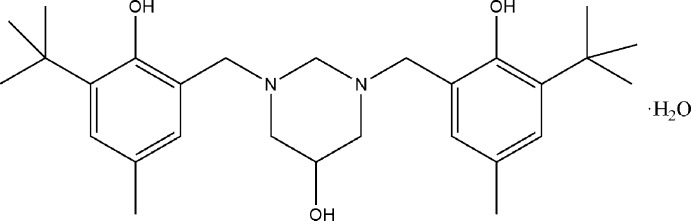


We have previously reported the synthesis and crystal structure of 1,3-bis­(3-*tert*-butyl-2-hy­droxy-5-meth­oxy­benz­yl)-1,3-diazinan-5-ol monohydrate (**II**) and this study has shown that the hydroxyl substituent on the 1,3-diazinane ring is disordered over two positions, namely one component equatorial and the other axial (Rivera *et al.*, 2014 ▸). As a logical step in the progression of these studies, in this paper we discuss the synthesis and crystal structure of the title compound (**I**), 1,3-bis­(3-*tert*-butyl-2-hy­droxy-5-methyl­benz­yl)-1,3-diazinan-5-ol monohydrate. The X-ray study again reveals that compound crystallizes with a solvent water mol­ecule that links to the organic mol­ecule through an O—H⋯O hydrogen bond. Furthermore, the hydroxyl group in the pyrimidine ring is also disordered over two positions (axial, equatorial).

## Structural commentary   

**Figure 1:**
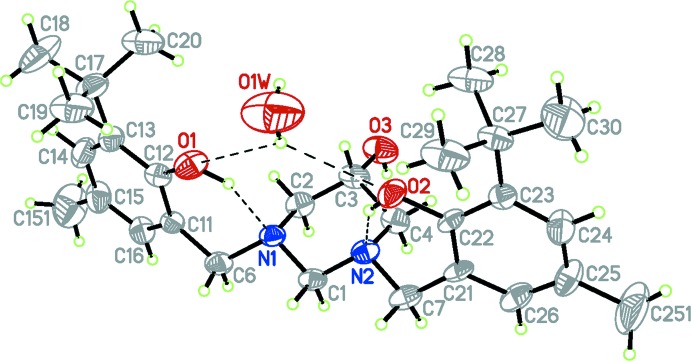
The mol­ecular structure of the title compound. Displacement ellipsoids are drawn at the 50% probability level. Hydrogen bonds are drawn as dashed lines and, for clarity, only the major-disorder component (equatorial) of the –OH substituent on the pyrimidine ring is included.

The mol­ecular structure of the title compound is presented in Fig. 1 ▸. The structure consists of a 1,3-bis­(3-*tert*-butyl-2-hy­droxy-5-methyl­benz­yl)-1,3-diazinan-5-ol mol­ecule and a water mol­ecule. These components are connected by an O3—H3⋯O1*W* hydrogen bond (Table 1 ▸) with the water-O atom as the acceptor. The 1,3-diazinane ring adopts a chair conformation with puckering parameters: *Q* = 0.588 (2) Å, θ = 176.9 (5) and *φ* = 245 (9)°. Atoms N1 and N2 are essentially tetra­hedral (bond-angle sums are 331.5° for N1 and 331.6° for N2), with their benzyl substituents in equatorial positions and the lone pairs axial. The aromatic rings of these substituents are roughly parallel, with a dihedral angle between the two benzene rings of 19.7 (4)°. Intra­molecular O—H⋯N hydrogen bonds form between the pyrimidine N atoms and the OH groups of the benzyl substit­uents and the pyrimidine N atoms, each with an *S*(6) graph-set motif (Table 1 ▸). These inter­actions stabilize the mol­ecular conformation, with O1⋯N1 = 2.696 (5) and O2⋯N2 = 2.702 (5) Å. These distances are closely comparable to those observed in the related structure (**II**) (Rivera *et al.*, 2014 ▸).

The N2—C7 distance of 1.485 (6) Å is slightly longer than the typical value for an N—C bond [1.469 Å]. The remaining C—N bonds in the mol­ecule are also typical and compare well with those found in the the related structure (**II**) (Rivera *et al.*, 2014 ▸). The C12—O1 and C22—O2 distances are typical of those for a hy­droxy substituent on an aromatic ring [1.376 (6) and 1.374 (5) Å, respectively]. Bond angles within the 1,3-diazinane ring are unexceptional. The hydroxyl group is disordered over two positions, with site occupancies refining to 0.794 (13) and 0.206 (13). The OH group of the major component is in the equatorial position with the minor component axial.

## Supra­molecular features   

**Figure 2:**
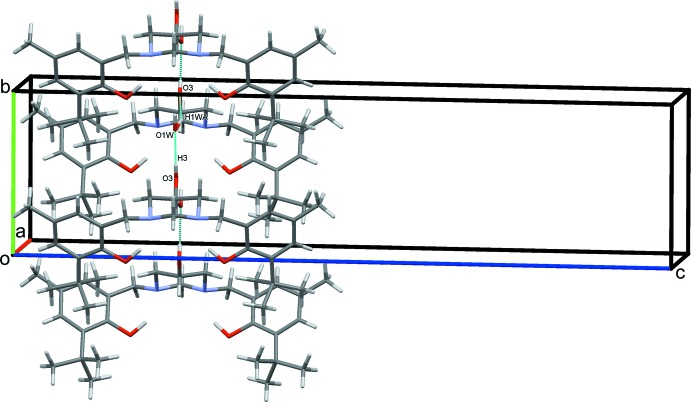
Part of the crystal packing of the title compound, showing the extensive inter­molecular hydrogen-bonding inter­actions (dashed lines). For clarity, only the major-disorder components (equatorial) of the OH substituents on the pyrimidine rings are included.

In the crystal, O3—H3⋯O1*W* hydrogen bonds form chains along *b*. These contacts are augmented by additional strong O1*W*—H1*WA*⋯O3 hydrogen bonds, this time with O3 as the acceptor (Fig. 2 ▸, Table 1 ▸). The chains are held together by van der Waals forces.

## Database survey   

Apart from the previously published structure (Rivera *et al.*, 2014 ▸), there is only one similar entry in the CSD (Mendes *et al.*, 2014 ▸). In this latter structure, the 1,3-diazinane mol­ecule acts as a ligand to an iron(III) cation, which would affect comparisons with the geometric parameters of the title compound.

## Synthesis and crystallization   

The title compound was prepared according to our reported method (Rivera *et al.*, 2016 ▸). The crude product was recrystallized from hexane solution, giving colorless crystals suitable for X-ray diffraction. M.p. 400 K, yield, 38%.

## Refinement   

Crystal data, data collection and structure refinement details are summarized in Table 2 ▸. The O3—H3 hydroxyl group is disordered over two positions, one with the OH group equatorial with the minor component axial. The site occupancies refine to 0.794 (13) and 0.206 (13), respectively. The H atom of the hydroxyl group of the major component was located in a difference map and refined freely while that of the minor component was fixed geometrically, both with *U*_iso_(H) set to 1.2*U*_eq_(O). The H atoms of the water mol­ecule were fixed in their found locations with *U*_iso_(H) set to 1.5*U*_eq_(O). C-bound H atoms were fixed geometrically (C—-H = 0.95 or 0.99 Å) and refined using a riding-model approximation, with *U*_iso_(H) set to 1.2*U*_eq_ of the parent atom. The crystal was a two-component twin with a fractional contribution to the minor domain of 0.0922 (18).

## Supplementary Material

Crystal structure: contains datablock(s) I. DOI: 10.1107/S2056989016013645/sj5503sup1.cif

Structure factors: contains datablock(s) I. DOI: 10.1107/S2056989016013645/sj5503Isup2.hkl

Supporting information file. DOI: 10.1107/S2056989016013645/sj5503Isup3.cml

CCDC reference: 1500903

Additional supporting information: crystallographic information; 3D view; checkCIF report

## Figures and Tables

**Table 1:** Hydrogen-bond geometry (Å, °)

*D*—H⋯*A*	*D*—H	H⋯*A*	*D*⋯*A*	*D*—H⋯*A*
O1—H1⋯N1	0.95 (7)	1.84 (7)	2.696 (5)	148 (5)
O2—H2⋯N2	0.96 (6)	1.81 (6)	2.702 (5)	152 (5)
O3—H3⋯O1*W* ^i^	0.76 (9)	2.12 (9)	2.882 (8)	177 (9)
O1*W*—H1*WA*⋯O3^ii^	0.94	1.98	2.873 (8)	158
O1*W*—H1*WA*⋯O3′^ii^	0.94	2.19	2.80 (2)	122
O1*W*—H1*WB*⋯O2	0.84	2.64	3.057 (7)	112

Symmetry codes: (i) 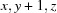; (ii) 
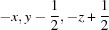
.

**Table 2:** Experimental details

Crystal data	
Chemical formula	C_28_H_42_N_2_O_3_·H_2_O
*M*_r_	472.65
Crystal system, space group	Monoclinic, *P*2_1_/*c*
Temperature (K)	173
*a*, *b*, *c* (Å)	10.11944 (9), 8.25445 (8), 33.8907 (3)
β (°)	97.8676 (4)
*V* (Å^3^)	2804.26 (4)
*Z*	4
Radiation type	Cu *K*α
μ (mm^−1^)	0.59
Crystal size (mm)	0.25 × 0.25 × 0.09

Data collection	
Diffractometer	Bruker APEXII CCD three-circle
Absorption correction	Multi-scan (*SADABS*; Bruker, 1998 ▸)
*T*_min_, *T*_max_	0.746, 1.000
No. of measured, independent and observed [*I* > 2σ(*I*)] reflections	25653, 3138, 2895
*R*_int_	0.053
θ_max_ (°)	51.7
(sin θ/λ)_max_ (Å^−1^)	0.509

Refinement	
*R*[*F* ^2^ > 2σ(*F* ^2^)], *wR*(*F* ^2^), *S*	0.079, 0.207, 1.07
No. of reflections	3138
No. of parameters	333
H-atom treatment	H atoms treated by a mixture of independent and constrained refinement
Δρ_max_, Δρ_min_ (e Å^−3^)	0.28, −0.31

Computer programs: *APEX2* (Bruker, 2004 ▸), *SAINT* (Bruker, 1998 ▸), *SHELXS97* (Sheldrick, 2008 ▸), *SHELXL2014* (Sheldrick, 2015 ▸) and *XP* in *SHELXTL-Plus* (Sheldrick, 2008 ▸).

## supplementary crystallographic information

### Crystal data

**Table:**

C_28_H_42_N_2_O_3_·H_2_O	*F*(000) = 1032
*M**_r_* = 472.65	*D*_x_ = 1.120 Mg m^−^^3^
Monoclinic, *P*2_1_/*c*	Cu *K*α radiation, λ = 1.54187 Å
*a* = 10.11944 (9) Å	Cell parameters from 9999 reflections
*b* = 8.25445 (8) Å	θ = 2–50°
*c* = 33.8907 (3) Å	µ = 0.59 mm^−^^1^
β = 97.8676 (4)°	*T* = 173 K
*V* = 2804.26 (4) Å^3^	Plate, colourless
*Z* = 4	0.25 × 0.25 × 0.09 mm

### Data collection

**Table:**

Bruker APEXII CCD three-circle diffractometer	2895 reflections with *I* > 2σ(*I*)
Radiation source: Incoatec microfocus source	*R*_int_ = 0.053
ω scans	θ_max_ = 51.7°, θ_min_ = 1.3°
Absorption correction: multi-scan (SADABS; Bruker, 1998)	*h* = −9→10
*T*_min_ = 0.746, *T*_max_ = 1.000	*k* = −7→8
25653 measured reflections	*l* = −34→34
3138 independent reflections	

### Refinement

**Table:**

Refinement on *F*^2^	0 restraints
Least-squares matrix: full	Hydrogen site location: mixed
*R*[*F*^2^ > 2σ(*F*^2^)] = 0.079	H atoms treated by a mixture of independent and constrained refinement
*wR*(*F*^2^) = 0.207	*w* = 1/[σ^2^(*F*_o_^2^) + (0.0797*P*)^2^ + 7.0703*P*] where *P* = (*F*_o_^2^ + 2*F*_c_^2^)/3
*S* = 1.07	(Δ/σ)_max_ < 0.001
3138 reflections	Δρ_max_ = 0.28 e Å^−^^3^
333 parameters	Δρ_min_ = −0.31 e Å^−^^3^

### Special details

**Table:**

Geometry. All esds (except the esd in the dihedral angle between two l.s. planes) are estimated using the full covariance matrix. The cell esds are taken into account individually in the estimation of esds in distances, angles and torsion angles; correlations between esds in cell parameters are only used when they are defined by crystal symmetry. An approximate (isotropic) treatment of cell esds is used for estimating esds involving l.s. planes.
Refinement. Refined as a 2-component twin.

### Fractional atomic coordinates and isotropic or equivalent isotropic displacement parameters (Å^2^)

**Table:**

	*x*	*y*	*z*	*U*_iso_*/*U*_eq_	Occ. (<1)
N1	0.3254 (4)	0.7763 (5)	0.21069 (11)	0.0316 (10)	
N2	0.3688 (4)	0.7733 (5)	0.28166 (11)	0.0328 (10)	
O1	0.2304 (4)	0.5043 (4)	0.17291 (11)	0.0455 (10)	
H1	0.255 (6)	0.578 (8)	0.1942 (19)	0.07 (2)*	
O2	0.3143 (3)	0.5008 (4)	0.32058 (10)	0.0400 (9)	
H2	0.329 (5)	0.575 (7)	0.2996 (17)	0.056 (17)*	
O3	0.0542 (5)	0.9917 (7)	0.25116 (16)	0.0540 (19)	0.794 (13)
H3	0.091 (8)	1.073 (11)	0.251 (2)	0.05 (3)*	0.794 (13)
O3'	0.087 (2)	0.732 (3)	0.2516 (8)	0.084 (10)	0.206 (13)
H3'	0.1248	0.6581	0.2401	0.126*	0.206 (13)
C1	0.4256 (4)	0.8013 (6)	0.24530 (13)	0.0316 (11)	
H1A	0.5012	0.7262	0.2441	0.038*	
H1B	0.4600	0.9135	0.2451	0.038*	
C2	0.2173 (5)	0.8935 (6)	0.21124 (14)	0.0375 (12)	
H2A	0.1483	0.8753	0.1880	0.045*	
H2B	0.2526	1.0047	0.2094	0.045*	
C3	0.1561 (5)	0.8758 (6)	0.24922 (15)	0.0379 (13)	
H3A	0.1156	0.7654	0.2495	0.046*	0.794 (13)
H3B	0.0914	0.9667	0.2503	0.046*	0.206 (13)
C4	0.2634 (5)	0.8907 (6)	0.28543 (14)	0.0370 (12)	
H4A	0.3007	1.0017	0.2870	0.044*	
H4B	0.2240	0.8695	0.3101	0.044*	
C6	0.3872 (5)	0.7878 (6)	0.17401 (14)	0.0378 (13)	
H6A	0.4694	0.7213	0.1772	0.045*	
H6B	0.4135	0.9017	0.1703	0.045*	
C7	0.4746 (5)	0.7809 (6)	0.31659 (14)	0.0387 (12)	
H7A	0.5061	0.8942	0.3202	0.046*	
H7B	0.5512	0.7140	0.3110	0.046*	
C11	0.2987 (4)	0.7334 (5)	0.13747 (14)	0.0308 (12)	
C12	0.2251 (5)	0.5891 (5)	0.13778 (14)	0.0310 (12)	
C13	0.1507 (5)	0.5278 (6)	0.10314 (15)	0.0377 (13)	
C14	0.1514 (6)	0.6207 (7)	0.06904 (15)	0.0485 (15)	
H14	0.1022	0.5821	0.0450	0.058*	
C15	0.2198 (6)	0.7672 (7)	0.06775 (15)	0.0490 (15)	
C16	0.2925 (5)	0.8205 (6)	0.10255 (15)	0.0405 (13)	
H16	0.3398	0.9199	0.1025	0.049*	
C17	0.0760 (5)	0.3666 (6)	0.10303 (17)	0.0499 (15)	
C18	0.0022 (8)	0.3268 (9)	0.0615 (2)	0.095 (3)	
H18A	−0.0621	0.4130	0.0530	0.143*	
H18B	0.0667	0.3184	0.0425	0.143*	
H18C	−0.0452	0.2236	0.0625	0.143*	
C19	0.1749 (6)	0.2298 (6)	0.1152 (2)	0.071 (2)	
H19A	0.1268	0.1267	0.1151	0.106*	
H19B	0.2399	0.2240	0.0963	0.106*	
H19C	0.2216	0.2508	0.1420	0.106*	
C20	−0.0288 (6)	0.3723 (7)	0.1317 (2)	0.0652 (18)	
H20A	−0.0921	0.4601	0.1239	0.098*	
H20B	−0.0767	0.2689	0.1308	0.098*	
H20C	0.0156	0.3915	0.1589	0.098*	
C21	0.4305 (4)	0.7238 (5)	0.35448 (14)	0.0292 (11)	
C22	0.3553 (4)	0.5812 (5)	0.35560 (13)	0.0259 (11)	
C23	0.3228 (4)	0.5197 (6)	0.39176 (13)	0.0307 (11)	
C24	0.3686 (6)	0.6071 (7)	0.42536 (15)	0.0469 (14)	
H24	0.3490	0.5673	0.4502	0.056*	
C25	0.4417 (6)	0.7497 (7)	0.42547 (16)	0.0535 (16)	
C26	0.4698 (5)	0.8058 (6)	0.38942 (15)	0.0431 (14)	
H26	0.5180	0.9043	0.3886	0.052*	
C27	0.2443 (5)	0.3604 (6)	0.39375 (15)	0.0370 (13)	
C28	0.1069 (5)	0.3741 (7)	0.36836 (19)	0.0559 (16)	
H28A	0.0569	0.4634	0.3783	0.084*	
H28B	0.1185	0.3950	0.3406	0.084*	
H28C	0.0577	0.2726	0.3700	0.084*	
C29	0.3219 (6)	0.2220 (6)	0.3783 (2)	0.0593 (17)	
H29A	0.3377	0.2457	0.3510	0.089*	
H29B	0.4075	0.2094	0.3954	0.089*	
H29C	0.2704	0.1215	0.3786	0.089*	
C30	0.2210 (8)	0.3202 (9)	0.4363 (2)	0.081 (2)	
H30A	0.1709	0.4084	0.4467	0.121*	
H30B	0.1699	0.2193	0.4363	0.121*	
H30C	0.3071	0.3072	0.4531	0.121*	
C151	0.2103 (9)	0.8663 (9)	0.03030 (18)	0.090 (2)	
H15A	0.1229	0.9194	0.0256	0.135*	
H15B	0.2211	0.7956	0.0077	0.135*	
H15C	0.2807	0.9487	0.0332	0.135*	
C251	0.4875 (10)	0.8412 (10)	0.4634 (2)	0.105 (3)	
H25A	0.4431	0.9469	0.4625	0.158*	
H25B	0.5843	0.8570	0.4660	0.158*	
H25C	0.4651	0.7793	0.4862	0.158*	
O1W	0.1841 (5)	0.3019 (6)	0.2497 (2)	0.120 (2)	
H1WA	0.1012	0.3526	0.2427	0.179*	
H1WB	0.2232	0.3920	0.2488	0.179*	

### Atomic displacement parameters (Å^2^)

**Table:**

	*U*^11^	*U*^22^	*U*^33^	*U*^12^	*U*^13^	*U*^23^
N1	0.027 (2)	0.028 (2)	0.040 (2)	−0.0053 (18)	0.0046 (18)	−0.0030 (18)
N2	0.031 (2)	0.029 (2)	0.037 (2)	−0.0030 (19)	−0.0001 (18)	0.0044 (18)
O1	0.052 (2)	0.030 (2)	0.053 (2)	−0.0120 (18)	0.0012 (18)	0.0074 (19)
O2	0.048 (2)	0.032 (2)	0.039 (2)	−0.0141 (17)	0.0033 (16)	−0.0058 (17)
O3	0.037 (3)	0.046 (4)	0.080 (4)	0.009 (3)	0.013 (3)	0.000 (3)
O3'	0.054 (15)	0.10 (2)	0.101 (18)	−0.040 (14)	0.025 (12)	−0.002 (15)
C1	0.027 (3)	0.024 (2)	0.044 (3)	−0.003 (2)	0.004 (2)	0.002 (2)
C2	0.034 (3)	0.031 (3)	0.046 (3)	0.005 (2)	0.000 (2)	0.003 (2)
C3	0.024 (3)	0.032 (3)	0.057 (3)	0.012 (3)	0.004 (2)	0.002 (2)
C4	0.034 (3)	0.029 (3)	0.048 (3)	0.004 (2)	0.009 (2)	0.002 (2)
C6	0.036 (3)	0.033 (3)	0.046 (3)	−0.011 (2)	0.010 (2)	0.000 (2)
C7	0.034 (3)	0.030 (3)	0.050 (3)	−0.006 (2)	−0.005 (2)	0.003 (2)
C11	0.030 (3)	0.019 (3)	0.044 (3)	−0.004 (2)	0.010 (2)	−0.006 (2)
C12	0.028 (3)	0.020 (3)	0.045 (3)	0.004 (2)	0.007 (2)	0.003 (2)
C13	0.035 (3)	0.026 (3)	0.052 (3)	0.002 (2)	0.005 (2)	−0.010 (2)
C14	0.052 (4)	0.051 (4)	0.040 (3)	0.001 (3)	−0.001 (3)	−0.014 (3)
C15	0.065 (4)	0.044 (4)	0.040 (3)	−0.002 (3)	0.014 (3)	−0.001 (3)
C16	0.051 (3)	0.030 (3)	0.043 (3)	−0.004 (3)	0.016 (3)	0.001 (2)
C17	0.038 (3)	0.036 (3)	0.073 (4)	−0.004 (3)	−0.001 (3)	−0.016 (3)
C18	0.106 (6)	0.072 (5)	0.100 (6)	−0.043 (5)	−0.015 (5)	−0.030 (4)
C19	0.046 (4)	0.021 (3)	0.148 (6)	−0.001 (3)	0.022 (4)	−0.017 (3)
C20	0.035 (3)	0.041 (4)	0.121 (5)	−0.004 (3)	0.016 (4)	−0.006 (3)
C21	0.022 (3)	0.018 (3)	0.044 (3)	−0.002 (2)	−0.007 (2)	−0.004 (2)
C22	0.023 (3)	0.017 (3)	0.035 (3)	0.004 (2)	−0.007 (2)	−0.008 (2)
C23	0.026 (3)	0.028 (3)	0.038 (3)	0.005 (2)	0.003 (2)	−0.002 (2)
C24	0.059 (4)	0.046 (3)	0.036 (3)	−0.003 (3)	0.009 (3)	−0.005 (3)
C25	0.068 (4)	0.045 (4)	0.044 (3)	−0.008 (3)	−0.002 (3)	−0.021 (3)
C26	0.046 (3)	0.027 (3)	0.053 (4)	−0.007 (3)	−0.006 (3)	−0.010 (3)
C27	0.027 (3)	0.030 (3)	0.055 (3)	0.001 (2)	0.010 (2)	0.005 (2)
C28	0.031 (3)	0.040 (3)	0.098 (5)	−0.004 (3)	0.014 (3)	0.003 (3)
C29	0.048 (4)	0.023 (3)	0.110 (5)	−0.003 (3)	0.022 (3)	0.002 (3)
C30	0.103 (6)	0.069 (5)	0.073 (4)	−0.024 (4)	0.023 (4)	0.014 (4)
C151	0.132 (7)	0.088 (5)	0.048 (4)	−0.009 (5)	0.010 (4)	0.016 (4)
C251	0.157 (8)	0.097 (6)	0.059 (4)	−0.050 (6)	0.006 (5)	−0.036 (4)
O1W	0.081 (4)	0.058 (3)	0.220 (7)	−0.002 (3)	0.022 (4)	−0.010 (4)

### Geometric parameters (Å, º)

**Table:**

N1—C1	1.456 (6)	C17—C20	1.535 (8)
N1—C2	1.462 (6)	C17—C18	1.536 (8)
N1—C6	1.469 (6)	C18—H18A	0.9800
N2—C1	1.448 (6)	C18—H18B	0.9800
N2—C4	1.460 (6)	C18—H18C	0.9800
N2—C7	1.485 (6)	C19—H19A	0.9800
O1—C12	1.376 (6)	C19—H19B	0.9800
O1—H1	0.95 (7)	C19—H19C	0.9800
O2—C22	1.374 (5)	C20—H20A	0.9800
O2—H2	0.96 (6)	C20—H20B	0.9800
O3—C3	1.415 (6)	C20—H20C	0.9800
O3—H3	0.76 (9)	C21—C26	1.375 (7)
O3'—C3	1.38 (2)	C21—C22	1.405 (6)
O3'—H3'	0.8400	C22—C23	1.407 (6)
C1—H1A	0.9900	C23—C24	1.374 (7)
C1—H1B	0.9900	C23—C27	1.542 (7)
C2—C3	1.510 (7)	C24—C25	1.390 (8)
C2—H2A	0.9900	C24—H24	0.9500
C2—H2B	0.9900	C25—C26	1.372 (8)
C3—C4	1.528 (7)	C25—C251	1.507 (8)
C3—H3A	1.0000	C26—H26	0.9500
C3—H3B	1.0000	C27—C29	1.519 (7)
C4—H4A	0.9900	C27—C30	1.529 (8)
C4—H4B	0.9900	C27—C28	1.536 (7)
C6—C11	1.494 (7)	C28—H28A	0.9800
C6—H6A	0.9900	C28—H28B	0.9800
C6—H6B	0.9900	C28—H28C	0.9800
C7—C21	1.493 (7)	C29—H29A	0.9800
C7—H7A	0.9900	C29—H29B	0.9800
C7—H7B	0.9900	C29—H29C	0.9800
C11—C16	1.378 (7)	C30—H30A	0.9800
C11—C12	1.406 (6)	C30—H30B	0.9800
C12—C13	1.400 (7)	C30—H30C	0.9800
C13—C14	1.388 (7)	C151—H15A	0.9800
C13—C17	1.530 (7)	C151—H15B	0.9800
C14—C15	1.397 (8)	C151—H15C	0.9800
C14—H14	0.9500	C251—H25A	0.9800
C15—C16	1.374 (7)	C251—H25B	0.9800
C15—C151	1.502 (8)	C251—H25C	0.9800
C16—H16	0.9500	O1W—H1WA	0.9381
C17—C19	1.528 (8)	O1W—H1WB	0.8447
			
C1—N1—C2	109.6 (4)	C20—C17—C18	107.2 (5)
C1—N1—C6	110.0 (3)	C17—C18—H18A	109.5
C2—N1—C6	111.9 (4)	C17—C18—H18B	109.5
C1—N2—C4	110.4 (4)	H18A—C18—H18B	109.5
C1—N2—C7	110.2 (4)	C17—C18—H18C	109.5
C4—N2—C7	111.0 (4)	H18A—C18—H18C	109.5
C12—O1—H1	108 (4)	H18B—C18—H18C	109.5
C22—O2—H2	106 (3)	C17—C19—H19A	109.5
C3—O3—H3	104 (6)	C17—C19—H19B	109.5
C3—O3'—H3'	109.5	H19A—C19—H19B	109.5
N2—C1—N1	110.5 (3)	C17—C19—H19C	109.5
N2—C1—H1A	109.6	H19A—C19—H19C	109.5
N1—C1—H1A	109.6	H19B—C19—H19C	109.5
N2—C1—H1B	109.6	C17—C20—H20A	109.5
N1—C1—H1B	109.6	C17—C20—H20B	109.5
H1A—C1—H1B	108.1	H20A—C20—H20B	109.5
N1—C2—C3	110.0 (4)	C17—C20—H20C	109.5
N1—C2—H2A	109.7	H20A—C20—H20C	109.5
C3—C2—H2A	109.7	H20B—C20—H20C	109.5
N1—C2—H2B	109.7	C26—C21—C22	118.9 (4)
C3—C2—H2B	109.7	C26—C21—C7	120.0 (4)
H2A—C2—H2B	108.2	C22—C21—C7	121.0 (4)
O3'—C3—C2	113.6 (11)	O2—C22—C21	118.8 (4)
O3—C3—C2	111.2 (4)	O2—C22—C23	119.9 (4)
O3'—C3—C4	109.2 (12)	C21—C22—C23	121.2 (4)
O3—C3—C4	110.5 (4)	C24—C23—C22	116.1 (4)
C2—C3—C4	110.4 (4)	C24—C23—C27	121.9 (4)
O3—C3—H3A	108.2	C22—C23—C27	122.0 (4)
C2—C3—H3A	108.2	C23—C24—C25	124.5 (5)
C4—C3—H3A	108.2	C23—C24—H24	117.8
O3'—C3—H3B	107.8	C25—C24—H24	117.8
C2—C3—H3B	107.8	C26—C25—C24	117.3 (5)
C4—C3—H3B	107.8	C26—C25—C251	120.8 (6)
N2—C4—C3	108.9 (4)	C24—C25—C251	121.9 (6)
N2—C4—H4A	109.9	C25—C26—C21	122.0 (5)
C3—C4—H4A	109.9	C25—C26—H26	119.0
N2—C4—H4B	109.9	C21—C26—H26	119.0
C3—C4—H4B	109.9	C29—C27—C30	108.3 (5)
H4A—C4—H4B	108.3	C29—C27—C28	109.5 (5)
N1—C6—C11	114.0 (4)	C30—C27—C28	107.4 (5)
N1—C6—H6A	108.8	C29—C27—C23	109.4 (4)
C11—C6—H6A	108.8	C30—C27—C23	111.9 (4)
N1—C6—H6B	108.8	C28—C27—C23	110.2 (4)
C11—C6—H6B	108.8	C27—C28—H28A	109.5
H6A—C6—H6B	107.7	C27—C28—H28B	109.5
N2—C7—C21	113.8 (4)	H28A—C28—H28B	109.5
N2—C7—H7A	108.8	C27—C28—H28C	109.5
C21—C7—H7A	108.8	H28A—C28—H28C	109.5
N2—C7—H7B	108.8	H28B—C28—H28C	109.5
C21—C7—H7B	108.8	C27—C29—H29A	109.5
H7A—C7—H7B	107.7	C27—C29—H29B	109.5
C16—C11—C12	119.1 (4)	H29A—C29—H29B	109.5
C16—C11—C6	120.4 (4)	C27—C29—H29C	109.5
C12—C11—C6	120.4 (4)	H29A—C29—H29C	109.5
O1—C12—C13	119.6 (4)	H29B—C29—H29C	109.5
O1—C12—C11	118.7 (4)	C27—C30—H30A	109.5
C13—C12—C11	121.6 (4)	C27—C30—H30B	109.5
C14—C13—C12	115.8 (4)	H30A—C30—H30B	109.5
C14—C13—C17	122.5 (5)	C27—C30—H30C	109.5
C12—C13—C17	121.7 (5)	H30A—C30—H30C	109.5
C13—C14—C15	124.3 (5)	H30B—C30—H30C	109.5
C13—C14—H14	117.8	C15—C151—H15A	109.5
C15—C14—H14	117.8	C15—C151—H15B	109.5
C16—C15—C14	117.3 (5)	H15A—C151—H15B	109.5
C16—C15—C151	121.0 (5)	C15—C151—H15C	109.5
C14—C15—C151	121.6 (5)	H15A—C151—H15C	109.5
C15—C16—C11	121.8 (5)	H15B—C151—H15C	109.5
C15—C16—H16	119.1	C25—C251—H25A	109.5
C11—C16—H16	119.1	C25—C251—H25B	109.5
C19—C17—C13	109.7 (4)	H25A—C251—H25B	109.5
C19—C17—C20	109.6 (5)	C25—C251—H25C	109.5
C13—C17—C20	110.8 (4)	H25A—C251—H25C	109.5
C19—C17—C18	107.9 (5)	H25B—C251—H25C	109.5
C13—C17—C18	111.5 (5)	H1WA—O1W—H1WB	90.3
			
C4—N2—C1—N1	−63.3 (5)	C151—C15—C16—C11	177.9 (6)
C7—N2—C1—N1	173.7 (4)	C12—C11—C16—C15	−2.2 (7)
C2—N1—C1—N2	62.5 (5)	C6—C11—C16—C15	175.2 (5)
C6—N1—C1—N2	−174.0 (4)	C14—C13—C17—C19	118.1 (6)
C1—N1—C2—C3	−58.2 (5)	C12—C13—C17—C19	−60.6 (6)
C6—N1—C2—C3	179.4 (4)	C14—C13—C17—C20	−120.7 (6)
N1—C2—C3—O3'	−68.0 (13)	C12—C13—C17—C20	60.6 (6)
N1—C2—C3—O3	178.0 (4)	C14—C13—C17—C18	−1.4 (7)
N1—C2—C3—C4	55.0 (5)	C12—C13—C17—C18	179.9 (5)
C1—N2—C4—C3	58.5 (5)	N2—C7—C21—C26	−139.0 (4)
C7—N2—C4—C3	−179.0 (4)	N2—C7—C21—C22	44.8 (6)
O3'—C3—C4—N2	70.9 (11)	C26—C21—C22—O2	179.0 (4)
O3—C3—C4—N2	−178.0 (4)	C7—C21—C22—O2	−4.8 (6)
C2—C3—C4—N2	−54.7 (5)	C26—C21—C22—C23	−1.5 (6)
C1—N1—C6—C11	169.1 (4)	C7—C21—C22—C23	174.7 (4)
C2—N1—C6—C11	−68.8 (5)	O2—C22—C23—C24	179.6 (4)
C1—N2—C7—C21	−169.3 (4)	C21—C22—C23—C24	0.1 (6)
C4—N2—C7—C21	68.0 (5)	O2—C22—C23—C27	1.5 (6)
N1—C6—C11—C16	137.4 (5)	C21—C22—C23—C27	−178.0 (4)
N1—C6—C11—C12	−45.3 (6)	C22—C23—C24—C25	0.8 (8)
C16—C11—C12—O1	−179.0 (4)	C27—C23—C24—C25	178.9 (5)
C6—C11—C12—O1	3.6 (6)	C23—C24—C25—C26	−0.2 (9)
C16—C11—C12—C13	3.0 (7)	C23—C24—C25—C251	179.2 (6)
C6—C11—C12—C13	−174.3 (4)	C24—C25—C26—C21	−1.3 (8)
O1—C12—C13—C14	−179.7 (4)	C251—C25—C26—C21	179.3 (6)
C11—C12—C13—C14	−1.8 (7)	C22—C21—C26—C25	2.2 (7)
O1—C12—C13—C17	−0.9 (7)	C7—C21—C26—C25	−174.1 (5)
C11—C12—C13—C17	177.0 (4)	C24—C23—C27—C29	−117.7 (5)
C12—C13—C14—C15	−0.3 (8)	C22—C23—C27—C29	60.2 (6)
C17—C13—C14—C15	−179.1 (5)	C24—C23—C27—C30	2.3 (7)
C13—C14—C15—C16	1.1 (8)	C22—C23—C27—C30	−179.7 (5)
C13—C14—C15—C151	−176.6 (6)	C24—C23—C27—C28	121.8 (5)
C14—C15—C16—C11	0.2 (8)	C22—C23—C27—C28	−60.2 (6)

### Hydrogen-bond geometry (Å, º)

**Table:**

*D*—H···*A*	*D*—H	H···*A*	*D*···*A*	*D*—H···*A*
O1—H1···N1	0.95 (7)	1.84 (7)	2.696 (5)	148 (5)
O2—H2···N2	0.96 (6)	1.81 (6)	2.702 (5)	152 (5)
O3—H3···O1*W*^i^	0.76 (9)	2.12 (9)	2.882 (8)	177 (9)
O1*W*—H1*WA*···O3^ii^	0.94	1.98	2.873 (8)	158
O1*W*—H1*WA*···O3′^ii^	0.94	2.19	2.80 (2)	122
O1*W*—H1*WB*···O2	0.84	2.64	3.057 (7)	112

Symmetry codes: (i) *x*, *y*+1, *z*; (ii) −*x*, *y*−1/2, −*z*+1/2.
